# Role of KIR3DS1 in human diseases

**DOI:** 10.3389/fimmu.2012.00326

**Published:** 2012-11-01

**Authors:** Christian Körner, Marcus Altfeld

**Affiliations:** Ragon Institute of Massachusetts General Hospital, Massachusetts Institute of Technology and Harvard UniversityCharlestown, MA, USA

**Keywords:** KIR3DS1, HLA, HIV-1, killer-immunoglobulin-like receptors, NK cell

## Abstract

The function of natural killer (NK) cells is controlled by several activating and inhibitory receptors, including the family of killer-immunoglobulin-like receptors (KIRs). One distinctive feature of KIRs is the extensive number of various haplotypes generated by the gene content within the *KIR* gene locus as well as by highly polymorphic members of the KIR gene family, namely *KIR3DL1/S1*. Within the *KIR3DL1/S1* gene locus, *KIR3DS1* represents a conserved allelic variant and displays other unique features in comparison to the highly polymorphic *KIR3DL1* allele. *KIR3DS1* is present in all human populations and belongs to the *KIR* haplotype group *B*. *KIR3DS1* encodes for an activating receptor featuring the characteristic short cytoplasmic tail and a positively charged residue within the transmembrane domain, which allows recruitment of the ITAM-bearing adaptor molecule DAP12. Although HLA class I molecules are thought to represent natural KIR ligands, and HLA-Bw4 molecules serve as ligands for KIR3DL1, the ligand for KIR3DS1 still needs to be identified. Despite the lack of formal evidence for an interaction of KIR3DS1 with HLA-Bw4-I80 or any other HLA class I subtype to date, a growing number of associations between the presence of *KIR3DS1* and the outcome of viral infections have been described. Especially, the potential protective role of *KIR3DS1* in combination with *HLA-Bw4-I80* in the context of HIV-1 infection has been studied intensively. In addition, a number of recent studies have associated the presence or absence of *KIR3DS1* with the occurrence and outcome of some malignancies, autoimmune diseases, and graft-versus-host disease (GVHD). In this review, we summarize the present knowledge regarding the characteristics of KIRD3S1 and discuss its role in various human diseases.

## INTRODUCTION

Killer-immunoglobulin (Ig)-like receptors (KIRs) represent a family of activating and inhibitory receptors expressed on natural killer (NK) cells that shape and regulate NK cell functions (Moretta et al., 1993). All human KIRs share common structural features including a cytoplasmic domain, a transmembrane domain and either two or three Ig-like extracellular domains (D0, D1, and D2). Each of these structural domains has a functional role with regards to ligand specificity and signal transduction after engagement of the respective receptor. Based on the number of extracellular domains, KIR proteins are classified as KIR2D (two domains) and KIR3D (three domains) receptors. KIR2D receptors can be further subdivided into a type I and type II group depending on the characteristics of the two extracellular domains. Type I KIR2D proteins, such as KIR2DL1-3 and KIR2DS1-5, exhibit a D2 and D1 domain. In contrast, *KIR2DL4* and *KIR2DL5* encode for type II KIR2D proteins lacking the D1 domain but comprising a D0 domain similar to the membrane-distal domain of KIR3D proteins.

The intracellular domains of KIRs feature either a short or a long cytoplasmic tail (S or L in the nomenclature, respectively). The long cytoplasmic tail contains two immune tyrosine-based inhibitory motifs (ITIM) which lead to the transduction of an inhibitory signal and thus defines inhibitory KIRs. In contrast, KIR genes encoding for activating receptors possess short cytoplasmic tails, which have a positively charged amino acid residue in their transmembrane region. This allows recruitment of the DAP12 adaptor molecule that contains an immunoreceptor tyrosine-based activating motif (ITAM) and transmits an activating signal. An exception is KIR2DL4, which contains only one ITIM within the long cytoplasmic tail and additionally possesses a charged residue, thus, making it capable of triggering both inhibitory and activating signals (Faure and Long, 2002).

Within the 14 described human KIRs, KIR3DS1 displays several unique features compared, in particular, to its inhibitory counterpart KIR3DL1. KIR3DS1 represents the only activating receptor with three extracellular domains. Moreover, in contrast to the highly polymorphic *KIR3DL1, KIR3DS1* is almost monomorphic (Norman et al., 2007; Parham et al., 2011). The interactions between KIR3DL1 and its natural ligands, HLA class I molecules of the HLA-Bw4 family have been studied intensively and confirmed by crystal structures (Vivian et al., 2011). In contrast, the natural ligand of KIR3DS1 has yet to be identified. Recent genetic and functional studies have suggested that certain HLA-Bw4 molecules containing an isoleucine in position 80 (HLA-Bw4-I80) are a potential putative ligand for KIR3DS1 (Martin et al., 2002; Alter et al., 2007, 2009). However, despite several attempts by different groups, direct interactions between KIR3DS1 and HLA-Bw4 molecules have not yet been demonstrated (Carr et al., 2007; Gillespie et al., 2007; O’Connor et al., 2007), with the exception of one report by Li et al. (2010) suggesting interactions between KIR3DS1 and the HLA-Bw4-T80 allotype HLA-B*2705. However, the study itself displayed some limitations, which include the lack of adequate negative controls to prove that binding of KIR3DS1 to the HLA/peptide complex was specific. Nevertheless, several studies have observed significant associations between the presence of *KIR3DS1* and the outcome of human disease, suggesting an undiscovered role of KIR3DS1 in modulating NK cell function (Martin et al., 2002; Lopez-Vazquez et al., 2005; Gabriel et al., 2010).

## EVOLUTION AND GENETICS

In humans, *KIR3DL1/S1* is clustered with other genes of the KIR family within a 100–200 kb region of the leukocyte receptor complex (LRC) on chromosome 19 (19q13.4; Trowsdale, 2001). The KIR locus can be divided into a centromeric and a telomeric part, which are flanked by so called framework genes (Wilson et al., 2000). The centromeric region is confined by *KIR3DL3 *and *KIR3DP1*, while the telomeric region of the KIR gene locus is enclosed by *KIR2DL4* and *KIR3DL2*. Within the region between these framework genes, the numbers and types of KIRs present within a given individual are highly variable resulting in extensive numbers of different haplotypes. The current KIR genes and the resulting haplotypes display a snapshot of the rapid evolution of the KIR gene locus that is driven by selection. The close proximity of the KIR genes within the centromeric and telomeric region and the organization of the *KIR* locus in a head-to-tail fashion probably facilitated gene expansion by duplication and recombination, and is reflected by the substantial linkage disequilibrium between KIRs (Gourraud et al., 2010), e.g., between *KIR3DS1* and *KIR2DS1* (Hollenbach et al., 2010). In addition, unequal crossover and generation of extended *KIR* haplotypes with multiple copies of an individual gene have been described for *KIR3DL1/S1 *and *KIR2DL4* (Martin et al., 2003; Williams et al., 2003).

KIR haplotypes can be generally distinguished into two groups, A**and**B, which not only reflect differences in their gene content and allelic variability but also their contribution to the susceptibility of various diseases (Uhrberg et al., 1997; Hsu et al., 2002; Marsh et al., 2003). Individuals encoding the *KIR3DS1* gene are considered to have the KIR haplotype B, which is defined by the presence of at least one of the following genes: *KIR2DL5*, *KIR2DS1*, *KIR2DS2*, *KIR2DS3*, *KIR2DS5*, and *KIR3DS1*. Haplotype A is defined by the absence of the above genes, respectively. In contrast to the KIR haplotype A, in which genetic variability is created by a broad allelic variance within each KIR gene, variability within B haplotypes is achieved by the presence or absence of most of the activating KIR genes and *KIR2DL5*. Hence, alleles of activating KIRs account only for a minority of KIR alleles (Marsh et al., 2003; Robinson et al., 2010). This suggests a general evolutionary concept in which allelic variance of activating KIRs is either limited by natural selection potentially due to increased occurrence of autoimmune diseases, or is not required because of the vast possible combinations resulting from gene arrangements. An alternative hypothesis is that the limited diversification of activating KIRs simply reflects the shorter time period since the first appearance of activating KIRs as compared to the ancestral inhibitory KIR proteins. Nevertheless, *KIR* haplotype group A as well as group B are present in all human population indicating an evolutionary advantage for the maintenance of both haplotype groups (Gendzekhadze et al., 2006, 2009).

The *KIR3DL1/S1 *gene locus displays a unique feature of containing reciprocal subsets of allotypes encoding either for the inhibitory receptor KIR3DL1 or the activating receptor KIR3DS1 (Campbell and Purdy, 2011). While *KIR3DL1 *allotypes exhibit a high rate of single nucleotide polymorphisms (SNPs), *KIR3DS1* is almost monomorphic (Norman et al., 2007; Parham et al., 2011). As a consequence, most of the approximately 70 known allotypes of *KIR3DL1/S1* account for *KIR3DL1*. Because of its opposing functional characteristic in comparison to KIR3DL1, KIR3DS1 was initially considered to be the product of a discrete gene (Valiante et al., 1997). However, further segregation studies identified *KIR3DS1* as a functional allele of the *KIR3DL1/S1* gene locus (Marsh et al., 2003; Traherne et al., 2010).

The evolutionary history of *KIR3DL1/S1* can be traced back before the radiation of the placental mammals, approximately 135 million ago. The ancestral founder gene *KIR3D* diverged upon gene duplication into *KIR3DX* and *KIR3DL *(Sambrook et al., 2006; Guethlein et al., 2007). While *KIR3DX* remained a pseudogene within the leukocyte Ig-like receptors (LILR) gene locus, *KIR3DL* expanded and evolved rapidly through duplication and recombination forming diverse lineages within simian primates. Based on ancestral inhibitory KIRs with their characteristic ITIMs located within the long cytoplasmic tail, the appearance of KIRs with activating properties resulted from new mutations within the TM and the cytoplasmic tail (Abi-Rached and Parham, 2005). Elimination of ITIMs that account for the suppressive properties of inhibitory KIRs resulted from mutations or stop codons leading to alterations of length of the cytoplasmic tail. This process was accompanied by the introduction of a positively charged amino acid residue within the TM allowing activating KIRs to interact with the DAP12 adaptor molecule, which possesses an ITAM (Lanier et al., 1998; Vivier et al., 2004).

Based on sequences as well as on the presence and frequency of *KIR3DL1/S1* allotypes in the human populations, *KIR3DL1/S1* alleles can be classified into three distinct phylogenetic lineages that evolved more than 3 million years ago: 3DL1*015-like, 3DL1*005-like, and 3DS1*013-like (Norman et al., 2007; Parham et al., 2011). In line with this, the allotypes KIR3DL1*015, KIR3DL1*005, and 3DS1*01301 each represent the prototypical alleles of the lineages due to their abundance in all human populations*.* Although KIR3DS1*01301 is the most abundant *KIR3DL1/S1* allele worldwide, lower allele frequencies of *KIR3DS1 *in sub-Saharan Africans have been described, indicating that natural selection for inhibitory alleles of *KIR3DL1/S1* is potentially due to increased exposure to a wide variety of pathogens over a long time period (Norman et al., 2007). In this context, Abi-Rached and Parham (2005) introduced a provocative hypothesis that might provide an explanation for the maintenance and diversification of activating KIRs as a result of balanced selection of the KIR haplotype groups A and B**(enriched for activating KIRs) observed in modern human populations. The authors postulated that activating KIRs undergo an initial phase of positive selection, which is driven by an increased resistance to pathogens and reproductive success. This phase is then followed by a period of negative selection that is caused by the negative effects of autoimmunity. Although the presence and maintenance of both KIR haplotype groups A and B in all human populations are in line with this hypothesis and indicate a complementary function of the KIR haplotype groups A and B, the underlying mechanisms are still under discussion. In particular, the epidemiological studies on KIR and reproduction that have suggested an association of maternal homozygosity for KIR haplotype group A with an increased risk for pregnancy complications such as preeclampsia and recurrent miscarriage are so far limited to KIR2D molecules (Hiby et al., 2004, 2008, 2010).

## REGULATION OF TRANSCRIPTION AND EXPRESSION

The expression patterns of human KIR proteins within a single individual’s NK cell population are highly complex. They may be affected by many factors including frequency, expression levels, and co-expression of other KIRs. These determinants are controlled by genetic and epigenetic factors some of which have been identified over the past years, in particular for *KIR3DL1/S1*. Key players in the regulation of KIR expression are the promoter regions, which display different affinities to transcription factors due to sequence variations.

Similar to most of the KIR genes, transcription of *KIR3DL1/S1* is regulated by a ~2 kb small region upstream of the coding region. With the exception of KIR2DL4, the promoters within this region are highly homologous throughout the KIR locus (Wilson et al., 2000). The expression of the encoded KIR genes is regulated by the degree of DNA methylation, which prevents transcription in the early stages of NK cell development (Chan et al., 2005; Santourlidis et al., 2008). During maturation and education of NK cells, the KIR expression pattern of each NK cell is thought to be defined by hypo- or hypermethylation of the distinct promoters (Santourlidis et al., 2002; Chan et al., 2003). The level of expression of *KIR3DL1/S1* is determined by a complex interplay of the activity of two promoters within the intergenic region upstream of *KIR3DL1/S1*. While the distal promoter only transcribes sense mRNA, the proximal promoter features a bidirectional promoter activity, producing either sense or antisense mRNA in a probabilistic fashion resulting in either silenced or increased expression of *KIR3DL1/S1 *(Stewart et al., 2003; van Bergen et al., 2005; Davies et al., 2007; Stulberg et al., 2007). SNPs within the proximal promoters determine the activity and transcription of sense and antisense mRNA, resulting in different expression levels and frequencies of the different *KIR3DL1/S1* alleles (Yawata et al., 2006; Li et al., 2008).

However, these SNPs can only partially explain the discordant observation of low transcription ratio and frequency of KIR3DS1 in NK cells. Depending on homo- or heterozygosity of the *KIR3DS1* allele, the frequency of NK cells expressing KIR3DS1 can range between ~ 40–80 and 10–60% respectively (O’Connor et al., 2007; Pascal et al., 2007). This indicates the involvement of other factors influencing KIR3DS1 expression on NK cells. In fact, it has been shown that surface expression of KIR3DS1 can be induced by various stimuli, such as IL-2, IL-15, or the MHC class I deficient K562 cell line (Morvan et al., 2009). Moreover, Yawata et al. (2006) observed increased numbers of NK cells expressing KIR3DL1 and KIR2DL1 in individuals carrying the cognate HLA ligands, indicating a possible effect of HLA ligands on the expression of their respective KIR receptors. Finally, frequency and surface density of KIR3DS1 can be further affected by copy number variants of *KIR3DL1/S1* within the KIR locus (Pelak et al., 2011). Increased RNA expression and percentage of KIR3DS1+ NK cells but not KIR3DL1+ NK cells were observed in individuals with multiple copies of *KIR3DL1* alleles, suggesting an interaction between the expression levels of these two allotypes that will require further investigations.

Surface expression of KIR3DS1 can be detected by the KIR3DL1/S1-specific mAb Z27 (Carr et al., 2007; O’Connor et al., 2007; Trundley et al., 2007). In combination with the KIR3DL1-specific mAb DX9 (Litwin et al., 1994), mAb Z27 is used to discriminate cells with regards to their surface expression of KIR3DL1 or KIR3DS1. While Z27 and DX9 antibodies both stain KIR3DL1, KIR3DS1 is not recognized by DX9, indicating that the antibodies target different epitopes. In line with this, it has been shown that Tyr^200^ is a determinant for DX9 recognition of KIR3DL1 but not for Z27 (Khakoo et al., 2002). Furthermore, different amino acid residues in position 199 of KIR3DL1 (proline) and KIR3DS1 (leucine) might contribute to the observed different staining profiles. KIR3DS1 displays low fluorescence intensity when stained with Z27. Whether this reflects a low binding affinity of Z27 to KIR3DS1 or an overall low surface expression of KIR3DS1 in comparison to KIR3DL1 is unclear. In contrast, KIR3DL1 displays various expression levels depending on the respective allotype, which can be discriminated into alleles with high (e.g., KIR3DL1*001) and low surface density (e.g., KIR3DL1*005), as well as a null variant (KIR3DL1*004) that is not expressed on the cell surface (Gardiner et al., 2001; Pando et al., 2003; Yawata et al., 2006). Of note, similar to the KIR3DL1*004 allele that encodes for a KIR3DL1 molecule that is not expressed on the cell surface, a *KIR3DS1*^null^ allele (KIR3DS1*049N) has also been identified (Pascal et al., 2007). A complex deletion/substitution mutation in exon 4 resulting in a premature stop codon was identified as the underlying mechanism. A subsequent study analyzing allele frequencies of KIR3DS1*049N in the different populations worldwide revealed that this allele is very rare, suggesting that KIR3DS1*049N is probably not importantly involved in disease associations described for *KIR3DS1 *(Martin et al., 2007a).

Despite these recent advances in our understanding of the regulation of KIR expression on a single cell level, the underlying mechanisms of how the KIR expression patterns are shaped are still not fully understood. In general, cytokines and the presence cognate HLA ligands may be involved during maturation and education of NK cells thus influencing KIR expression patterns within the NK cell population. With regards to KIR3DS1, further studies are needed to fully understand how expression of KIR3DS1 is regulated and to determine the potential functional consequences of increased expression.

## LIGAND SPECIFICITY OF KIR3DL1 AND KIR3DS1

Classical and non-classical HLA class I molecules can serve as natural ligands for several inhibitory and activating KIRs (Biassoni et al., 1995; Rajagopalan and Long, 1999; Hansasuta et al., 2004). Moreover, crystal structures of certain KIR/peptide/HLA complexes have clarified the molecular interactions between KIR and peptide-loaded HLA molecules (Boyington et al., 2000; Fan et al., 2001; Vivian et al., 2011), including the KIR3DL1 and HLA-B57 pair.

KIR3DL1 recognizes HLA-A and -B molecules containing the HLA-Bw4 motif (Cella et al., 1994; Gumperz et al., 1995; Stern et al., 2008). However, KIR3DL1 displays differential affinities to its cognate HLA class I ligands depending on the encoding KIR3DL1 allele, the respective HLA allele, and the HLA class I-presented epitope (Carr et al., 2005; Thananchai et al., 2007; Foley et al., 2008; Fadda et al., 2011). Minor changes in the amino acid sequence of KIR3DL1 in regions interacting with the peptide/HLA complex can lead to either increased affinity or complete abrogation of binding. Two polymorphisms at position 238 (D2 domain) and 320 (TM) that discriminate KIR3DL1*002 and KIR3DL1*007 were identified to affect the ability of HLA-Bw4 ligands to inhibit KIR3DL1*002- and KIR3DL1*007-expressing NK leukemia cell lines (Carr et al., 2005). Moreover, it has been shown that the differential binding of KIR3DL1 to HLA-A*2402 in complex with an HIV-1 peptide is mediated by dimorphic motifs in the D0 and D1/D2 domains of KIR3DL1 (Sharma et al., 2009). Thus, single amino acid substitutions within KIR3DL1 play a critical role in the binding affinity to HLA ligands and can provide insights into the molecular mechanisms that regulate KIR/HLA interactions. In addition, it has been shown that a single amino acid change in the KIR2D receptors defines the ability of KIR2D receptors to recognize either HLA-C group 1 or 2 receptors. This suggests a unifying concept of ligand specificity and affinity for KIR proteins (Winter and Long, 1997; Moesta et al., 2008).

The binding affinity of KIR3DL1 to its cognate HLA class I molecules is further dependent on the HLA class I presented peptides. In particular, amino acid residues in position 7 and 8 of the HLA-bound peptide seem to be pivotal in either promoting or abrogating binding of KIRs to their cognate HLA class I molecules (Peruzzi et al., 1996; Mandelboim et al., 1997; Rajagopalan and Long, 1997; Vales-Gomez et al., 1998; Hansasuta et al., 2004; Fadda et al., 2010). In this context, it has been shown that binding of KIR3DL1 to HLA-B57 was affected by a variety of HIV-1-derived peptides (Fadda et al., 2011). Crystal structures of KIR/HLA/peptide complexes confirmed a direct interaction between residues 7 and 8 of the HLA-presented peptide and KIRs (Boyington et al., 2000; Vivian et al., 2011). Overall, amino acid polymorphisms within KIR proteins and HLA class I-presented epitopes represent two pivotal mechanisms defining the binding of KIR proteins to their cognate HLA ligands. These polymorphisms might provide NK cells with an ability to distinguish changes of the epitope repertoire presented by HLA class I on virus-infected and transformed cells.

Despite a high sequence homology (>95%) between *KIR3DS1* and *KIR3DL1 *(Dohring et al., 1996), direct binding of KIR3DS1 to HLA class I molecules has not been convincingly demonstrated to date. However, several genetic studies have suggested a functional interaction between KIR3DS1 and HLA-Bw4 molecules in human diseases (Martin et al., 2002; Lopez-Vazquez et al., 2005). Martin et al. (2002) first identified an association between *KIR3DS1* and certain *HLA-B* alleles and the progression to AIDS. The combined presence of *KIR3DS1* and *HLA-B* alleles that encode an isoleucine at position 80 (HLA-Bw4-I80) was associated with significantly slower progression to AIDS, while the absence of either *KIR3DS1* or *HLA-Bw4-I80* alleles led to the loss of the observed effect. The combined *KIR3DS1/HLA-Bw4-I80 *genotype was also associated with decreased susceptibility for the development of hepatocellular carcinoma in patients with chronic hepatitis C virus (HCV; Lopez-Vazquez et al., 2005). These data implicate a potential interaction between HLA-Bw4-I80 molecules and KIR3DS1, and suggested a functional role for KIR3DS1 in NK cell-mediated recognition of HIV-1-infected as well as transformed cells. However, not all studies have demonstrated a synergistic protective effect of the compounded genotype in the context of HIV-1 infection. Other groups have shown that the beneficial effects of *HLA-Bw4-I80 *and *KIR3DS1* are independent and not linked to each other (Barbour et al., 2007), or have observed a detrimental effect of carriage of KIR3DS1 on progression of HIV-1 infection (Gaudieri et al., 2005).

Based on these genetic associations studies, several groups have sought to assess a direct interaction of KIR3DS1 and HLA-Bw4-I80 class I molecules. Gillespie et al. (2007) used multiple Bw4-I80 MHC class I tetramers refolded with a broad panel of HIV-1 peptide epitopes to detect tetramer binding to KIR3DS1 transiently expressed on 293-T cells, while O’Connor et al. (2007) used a luciferase reporter assay to detect activation of KIR3DS1-transfected Jurkat cells in response to stimulation with 721.221 cells transfected with multiple HLA-Bw4 molecules. Although these methods were successfully used to identify HLA/peptide complexes as ligands for KIR3DL1, the same assays failed to show any detectable interaction with KIR3DS1. Another approach was chosen by Carr et al. (2007) using KIR3DS1–IgG fusion constructs to identify binding with HLA-B*5701 transfected 721.221 cells. Again, the authors were not able to identify HLA-Bw4-I80 molecules as a ligand for KIR3DS1. In contrast to KIR3DL1+ NK cells, proliferation and cytotoxic capacity of KIR3DS1+ NK cells were not affected by HLA-Bw4^+^ irradiated allogeneic EBV-B cell lines (Morvan et al., 2008, 2009). The one exception is a recent study by Li et al. (2010) suggesting interactions between KIR3DS1 and HLA-Bw4-T80 allotype HLA-B*2705 by using surface plasmon resonance (SPR) technology. However, there were some limitations in the design of these studies, including the lack of negative controls that demonstrate that binding of KIR3DS1 to the HLA/peptide complex is specific. Overall, additional studies will be needed to determine whether KIR3DS1 can bind to HLA-Bw4 molecules, and whether these interactions depend on the HLA class I-presented epitope.

These studies which have investigated the allele-specific characteristics of KIR3DL1 have provided helpful insights in the potential molecular interaction between KIR3DS1 and HLA-Bw4 molecules (see **Table 1**). Despite the high level of homology to KIR3DL1, KIR3DS1 differs in at least six amino acid positions throughout the extracellular domains, including positions 163, 166 (D1 domain), and 199 (D2 domain; Gardiner et al., 2001). Introduction of any of these KIR3DS1-specific amino acid residues into a KIR3DL1*001 molecule as performed by Sharma et al. (2009) led to complete abrogation of binding to HLA-A*2402, a confirmed ligand for KIR3DL1. In this context, Thomas et al. (2008) identified a natural KIR3DL1 allotype (KIR3DL1*054) that shares the same amino acid residues at position 138, 163, and 166 of the D1 domain with KIR3DS1*013. Introduction of these single amino acid substitutions at position 138, 163, and 166 into KIR3DL1*01502 also lead to loss of recognition of HLA-A*2402 or HLA-B*2705 (O’Connor et al., 2011) confirming the importance of these residues. Moreover, the reverse introduction of KIR3DL1-specific residues in KIR3DS1*013 led to measurable binding of HLA-A*2402 but also revealed a complex interaction between distinct KIR3DS1-specific residues and HLA class I residues. While the substitution of tryptophan to glycine at position 138 seemed to be sufficient to reconstitute HLA binding, the additional introduction of proline at position 199 resulted in the loss of HLA binding. Interestingly, the existence of one *KIR3DS1* allotype (3DS1*014) carrying glycine at position 138 has been described previously (Crum et al., 2000; Norman et al., 2007). This potentially represents an intermediate step in the evolution of the activating KIR3DS1 from the ancestral inhibitory KIR. The extremely low frequency of this particular HLA-Bw4 binding KIR3DS1 allotype might reflect the strong negative effect on selection in the presence of a *KIR3DS1* allele with broad binding affinity to HLA-Bw4 molecules.

**Table 1:** Effects of selected amino acid variations within KIR3DL1/S1 on antibody recognition and HLA binding.

Domain	Position	3DS1^a^	3DL1^a^	Effect on antibody staining and HLA binding^b^	Reference
D0	58	G	S	DX9 and Z27 binding: reduction^c^	Sharma et al. (2009)
D0	92	M	V	DX9 and Z27 binding: reduction^c^	Sharma et al. (2009)
D1	138	W	G	DX9 and Z27 binding: no effect^c^, reduction^c^	Sharma et al. (2009), O’Connor et al. (2011)
				Tetramer binding (HLA-B*5701): no effect^d^	Vivian et al. (2011)
				Tetramer binding (HLA-A*2402): reduction^c^	O’Connor et al. (2011)
				Tetramer binding (HLA-B*2705): abrogation^c^	O’Connor et al. (2011)
D1	163	S	P	DX9 and Z27 binding: reduction^c^, no effect^c^	Sharma et al. (2009), O’Connor et al. (2011)
				Tetramer binding (HLA-A*2402): abrogation^c^, reduction^c^	Sharma et al. (2009), O’Connor et al. (2011)
				Tetramer binding (HLA-B*2705): abrogation^c^	O’Connor et al. (2011)
D1	166	R	L	DX9 binding: reduction^c^	Sharma et al. (2009)
				Tetramer binding (HLA-A*2402): abrogation^c^	Sharma et al. (2009), O’Connor et al. (2011)
				Tetramer binding (HLA-B*5701): reduction^d^	Vivian et al. (2011)
				Tetramer binding (HLA-B*2705): abrogation^c^	O’Connor et al. (2011)
D2	199	L	P	DX9 and Z27 binding: no effect^c^	Sharma et al. (2009)
				Tetramer binding (HLA-A*2402): abrogation^c^	Sharma et al. (2009)
				Tetramer binding (HLA-B*5701): reduction^d^	Vivian et al. (2011)

a Amino acid residue of KIR3DS1*013 and KIR3DL1*015, respectively;

b Tetramers folded with the following peptides: HLA-B*5701: LSSPVTKSF (self-peptide), HLA-A*2402: RYPLTFGW (HIV nef), HLA-B*2705: TSTLQEQIGW (HIV gag);

c Effect observed when aa introduced in KIR3DL1*015;

d Effect observed when aa introduced in KIR3DL1*001.

The crucial role of these amino acid residues within KIR3DL1 and KIR3DS1 in HLA binding was also confirmed by the recent description of the crystal structure of KIR3DL1*001 in complex with self-peptide-loaded HLA-B*5701 (Vivian et al., 2011). The authors identified a “hotspot” within the D1/D2 domain of KIR3DL1 that contains three loops (165–167, 199–201, and 278–282), which seem to be pivotal for binding to HLA-B57. This region is altered in KIR3DS1 and might provide an explanation for why there is no direct interaction between HLA-B*5701 and KIR3DS1. Whether the KIR3DS1-specific amino acid residues preclude recognition of other HLA-Bw4-I80 molecules is unknown and needs to be clarified by additional studies.

It has been shown that binding of KIR to its cognate ligand is dependent on the presented peptide (Malnati et al., 1995; Zappacosta et al., 1997; Maenaka et al., 1999; Stewart et al., 2005; Thananchai et al., 2007) thus raising the possibility that KIR3DS1 might bind HLA-Bw4-I80 molecules under certain circumstances. KIR3DL2 recognizes HLA-A*3 and HLA-A*11 molecules only in the presence of a small number of specific EBV-derived peptides (Hansasuta et al., 2004). Hence, KIR3DS1 might recognize HLA-Bw4 molecules in combination with specific peptides in a very restricted fashion. This would prohibit recognition and killing of self-peptide presenting normal cells and prevent autoimmune reactions of KIR3DS1+ NK cells. In contrast, it has been shown that stressed or virus-infected cells undergo alteration in their presented peptide repertoire (Hickman et al., 2003; Meiring et al., 2006). It is conceivable that these cells could be detected and killed by NK cells via engagement of activating KIRs such as KIR3DS1. However, the controversial discussion as to whether KIR3DS1 recognizes HLA-Bw4 molecules will continue until a direct interaction is proven. Thus, alternative hypotheses for effects exerted by *KIR3DS1* on the outcome of certain diseases continue to be a topic of interest to the field.

## ROLE OF THE KIR3DS1 IN DISEASES

Several studies have identified *KIR3DS1* to be associated with the outcome of various diseases (reviewed in Jamil and Khakoo, 2011), including viral infections, most prominently HIV-1 infection, certain malignancies, and auto immune diseases (see **Table 2**). In particular, the role of *KIR3DL1/S1* in viral infections has been well-accepted since the first descriptions of significant associations between the *KIR3DL1/S1 *gene locus and the outcome of HIV-1 infection (Martin et al., 2002, 2007b). Subsequent studies observed a protective effect of the combined presence of *KIR3DS1* and *HLA-Bw4-I80 *alleles in patients with chronic HIV-1 infection. These findings include key determinants of progressive HIV-1 infection such as lower viral load, slower decline of CD4+ T cell count and delayed progression to AIDS (Martin et al., 2002; Qi et al., 2006). In addition, in these studies it has been shown that an increased *KIR3DS1* count generated by copy number variants of *KIR3DL1/S1* was associated with a lower viral set point in the presence of *HLA-Bw4-I80 *alleles (Pelak et al., 2011).

**Table 2:** Selected disease associations of *KIR3DS1*.

Disease	Association when *KIR3DS1* is present
**Viral infections**	
HIV-1	Delayed progression to AIDS in combination with *HLA-Bw4-I80* (Martin et al., 2002)
Hepatitis B virus infection	Increased rate of spontaneous recovery (Zhi-Ming et al., 2007)
H1N1 influenza A	Severe pandemic influenza A infections (Aranda-Romo et al., 2012)
**Malignancies**	
Hepatocellular carcinoma	Reduced risk in combination with *HLA-Bw4-I80* (Lopez-Vazquez et al., 2005)
Hodgkin’s lymphoma	Reduced risk (Besson et al., 2007)
Respiratory papillomatosis	Reduced risk (Bonagura et al., 2010)
Cervical neoplasia	Increased risk (Carrington et al., 2005)
**Other**	
Allogeneic HSCT	Decreased progression-free survival of patients with multiple myeloma (Gabriel et al., 2010); decreased overall survival in *HLA-Bw4*^−^ patients (Gagne et al., 2009); decreased acute GVHD (Venstrom et al., 2010)
Ankylosing spondylitis (AS)	Increased susceptibility for development of AS (Lopez-Larrea et al., 2006; Diaz-Pena et al., 2010)

However, other observations questioned the concept of a synergistic effect of the *KIR3DS1*/*HLA-Bw4-I80 *compound genotype on HIV-1 disease, or the protective effect of *KIR3DS1* on the course of HIV-1 infection. A cohort study conducted by Barbour et al. (2007) indicated that* KIR3DS1* and *HLA-Bw4-I80* might independently exert a protective effect on the course of HIV-1 infection. The authors showed that HIV-1-positive individuals carrying *KIR3DS1* displayed increased CD4+ T cell counts as compared to those without *KIR3DS1* and that carriage of *HLA-Bw4-I80* was associated with lower viral loads. Gaudieri et al. (2005) reported a detrimental effect of the *KIR3DS1* allele which was associated with an increased hazard ratio for AIDS disease progression.

Another complicating issue is the inconsistency of findings in HIV-1 exposed seronegative subjects (HESN) regarding the influence of KIR3DS1 on HIV-1 acquisition. Two studies described a protective role of *KIR3DS1 *in these particular cohorts (Boulet et al., 2008; Tomescu et al., 2011). In contrast, two studies conducted by Tomescu et al. (2010) and O’Connell et al. (2009) did not observe an enrichment of *KIR3DS1* and *HLA-Bw4-I80* alleles in HESN (Tomescu et al., 2010). This suggests that host factors other than *KIR3DS1* may be involved in the control of HIV-1 infection.

Nevertheless, functional studies in KIR3DS1+ NK cells, in particular in acute HIV-1 infection, have provided some indications for an interaction of KIR3DS1 and HLA-Bw4-I80. Results obtained by Alter et al. (2009) demonstrated an expansion of KIR3DS1+ NK cells in acute HIV-1 infection, however only in individuals that also encoded for *HLA-Bw4-I80 *alleles. In addition, HLA-Bw4-I80-dependent killing of HIV-1 infected target cells and inhibition of viral replication by KIR3DS1+ NK cells has been described (Alter et al., 2007). Although a subsequent study conducted by Long et al. (2008) observed increased IFN-y and CD107a expression of NK cells in *KIR3DS1*+ individuals with early HIV-1 infection, this finding was independent of the joint carriage of *HLA-Bw4-I80.* However, the authors did observe higher IFN-y and CD107a expression in individuals encoding for both KIR3DS1 and HLA-Bw4 alleles, in particular in the presence of the B*58 alleles, and the lack of detecting a statistical significance for higher NK cell activity in KIR3DS1/*HLA-Bw4-I80*+**individuals might have been a consequence of the small sample size. In addition, increased frequency and antiviral capacity against HIV-1 of KIR3DS1+ NK cells were recently described in individuals encoding for multiple copies of *KIR3DL1 *(Pelak et al., 2011). The results from the study by Pelak et al. (2011) indicate that KIR3DL1-dependent licensing of NK cells might be involved in shaping a strong antiviral response of KIR3DS1+ NK cells. Higher responsiveness of licensed NK cells might therefore serve as one potential underlying mechanism for the protective effect of the combined presence of *KIR3DL1* and its cognate HLA ligands in chronic HIV-1 infection (Kim et al., 2008).

Overall, although a beneficial effect of *KIR3DS1* in the context of HIV-1 infection has been observed in some *in vivo* and *in vitro*, it remains unknown whether this effect is mediated by *KIR3DS1* itself or other genes in linkage disequilibrium with *KIR3DS1*. Furthermore, it is not clear whether changes in *KIR3DL1* copy numbers in *KIR3DS1*+ individuals are responsible for this protective effect or whether this is dependent on the simultaneous presence of *HLA-Bw4-I80*.

The role of *KIR3DS1* has also been investigated in other viral infections such as human T-cell leukemia virus type 1 (HTLV-1), H1N1 influenza A, and hepatitis B virus infection (HBV). While no evidence for the participation of *KIR3DS1 *in control of HTLV-1 infection was found (O’Connor et al., 2012), carriage of *KIR3DS1* and other haplotype B containing KIRs was associated with severe pandemic influenza A (H1N1) 2009 infections (Aranda-Romo et al., 2012). Frequencies of *KIR3DS1* as well as *KIR2DS1* and *KIR2DL5* were furthermore increased in a group of patients who spontaneously recovered from HBV compared to individuals with chronic HBV infection or healthy controls (Zhi-Ming et al., 2007). Because of the close proximity of the genes within the *KIR* locus and the resulting strong linkage disequilibrium with neighboring genes, it remains unclear whether the observed effects are due to a specific KIR. Furthermore, the mechanisms underlying a potential protective effect of specific KIR genotypes remain to be elucidated.

Aside from their important role in controlling viral infections, NK cells can also mediate antitumor immunity (reviewed in Wu and Lanier, 2003). Association studies investigating the impact of KIR/HLA haplotypes in selected malignancies have identified protective as well as unfavorable KIR/HLA haplotypes, including some comprising *KIR3DS1 *(Arnheim et al., 2005; Purdy and Campbell, 2009; Karabon et al., 2011). A protective effect of *KIR3DS1* in combination with HLA class I molecules expressing the *HLA-Bw4-I80* epitope in hepatocellular carcinoma has been observed in patients with chronic HCV infection (Lopez-Vazquez et al., 2005). The underlying mechanism of this protective genotype is not known. It is possible that transformed cells in the HCV-infected liver encode for a KIR3DS1 ligand and are directly recognized and killed by KIR3DS1+ NK cells. A decreased risk for the development of Hodgkin’s lymphoma (HL), a common lymphoma in young adults, was also identified in individuals carrying *KIR3DS1* in familial study with 90 cases and 255 first-degree relatives (Besson et al., 2007). In addition, absence of *KIR3DS1* and *KIR2DS1*, which display strong linkage disequilibrium, was associated with more frequent occurrence of respiratory papillomatosis (RRP), a rare disease of the larynx and upper airway caused by human papillomaviruses (HPV)-6/11 (Bonagura et al., 2010). Of note, all three malignancies described above are caused or at least promoted by viral infections, indicating a potential role of KIR3DS1 in the control of the underlying viral infections. Thus, it is possible that the protective role of KIR3DS1 is due to the recognition of virus-infected cells by KIR3DS1+ NK cells similar to other viral infections. In contrast, some studies have described a detrimental effect of *KIR3DS1 *in cervical cancer and on the outcome of unrelated hematopoietic stem cell transplantation (HSCT). An increased susceptibility to the development of cervical neoplasia in patients carrying *KIR3DS1* was identified by Carrington et al. (2005). Of note, similar to the above mentioned RRP, cervical neoplasia is also a HPV-associated disease. However, in this case the presence of KIR3DS1+ NK cells might be detrimental due to incomplete viral control and chronic inflammation that might facilitate the development of cancer.

In a multivariate analysis Gabriel et al. (2010) showed that progression-free survival of patients with multiple myeloma after autologous stem cell transplantation was significantly decreased in patients carrying the *KIR3DS1* gene. Furthermore, Gagne et al. (2009) observed the lowest overall survival in *HLA-Bw4*^−^ patients with myelogenous diseases receiving *KIR3DL1*^+^/KIR3DS1^+^ donor NK cells during HSCT. In contrast, a beneficial effect of *KIR3DS1* in the context of T cell-depleted HSCT has also been observed, showing a protective effect of donor KIR haplotype B against leukemic relapse and improved disease-free survival in patients undergoing HSCT (Verheyden et al., 2005; Marcenaro et al., 2011). Venstrom et al. (2010) furthermore showed that transplantation of hematopoietic stem cells from *KIR3DS1*^+^ donors was associated with decreased acute graft-versus-host disease (GVHD). Although the underlying mechanism of the observed effects are not understood, the above studies indicate a potential role of NK cell licensing in the setting of HSCT as the HLA and KIR haplotypes of the donor define NK cell responses in the new host.

Finally, with regards to autoimmune diseases, a growing number of studies suggest a role for NK cells in disease pathogenesis. In particular, *KIR3DS1* has been associated with the development and progression of ankylosing spondylitis (AS; reviewed in Diaz-Pena et al., 2009 and Azuz-Lieberman et al., 2005). AS represents a prototypic subtype of a group of various related diseases summarized as spondyloarthritis (reviewed in Dougados and Baeten, 2011). The characteristic symptoms include chronic inflammation of the joints and ligaments of the spinal bones that can be triggered by mechanical or bacterial stress. Although the exact underlying pathology remains unknown, genetic predisposition accounts for 80–90% of the cases, and include the presence of HLA-B27 allotypes as a major risk factor (Thomas and Brown, 2010). Several studies observed enrichment for *KIR3DS1* in *HLA-B27*+ patients with AS (Lopez-Larrea et al., 2006; Diaz-Pena et al., 2010), while *KIR3DL1* was found to be underrepresented in patients with AS as compared to *HLA-B27*+ healthy controls (Mousavi et al., 2010; Wang et al., 2012). With regards to the ligand specificity of KIR3DS1, functional studies investigating a potential interaction of KIR3DS1 and HLA-B27 in the context of AS have not been conducted. Moreover, whether the underlying mechanism of the increased susceptibility to the development of AS in *KIR3DS1*+ individuals involves a differential activation threshold of KIR3DS1+ NK or CD8+ T cells is not known. These data also indicate that the presence of *KIR3DS1* is a more important factor in the development of AS than the absence of protective KIR3DL1 allotypes. However, it should be noted that other studies neither observed any significant involvement of *KIR3DL1/S1* in the outcome of AS (Harvey et al., 2009), nor identified other compound KIR genes and HLA allotypes linked to the development and progression of AS (Diaz-Pena et al., 2008; Jiao et al., 2010; Tajik et al., 2011).

Taken together, these studies suggest that KIR3DS1 might not only play a role in the outcome of viral infections, but also in malignancies, the outcome after HSCT, and auto immune disease. Additional functional studies of NK cells are required in these disease settings to determine whether *KIR3DS1 *exerts its protective or detrimental effects through modulation of NK cell function, or by alternative mechanisms.

## CONCLUDING REMARKS

An accumulating number of studies have demonstrated the influence of *KIR3DS1* on the outcome of various human diseases. Furthermore, the ubiquitous presence of *KIR3DS1* within human populations suggests an evolutionary pressure to maintain this activating KIR. The lack of a defined ligand for KIR3DS1 has significantly hampered functional studies to determine the mechanisms underlying KIR3DS1-associated protection from disease, but might also suggest a restricted engagement of KIR3DS1 to prevent killing of normal cells and thus damage of the host.

## Conflict of Interest Statement

The authors declare that the research was conducted in the absence of any commercial or financial relationships that could be construed as a potential conflict of interest.
